# Dental anxiety in patients with borderline intellectual functioning and patients with intellectual disabilities

**DOI:** 10.1186/s12903-016-0312-y

**Published:** 2016-11-03

**Authors:** Antonio Fallea, Rosa Zuccarello, Francesco Calì

**Affiliations:** 1 Unit of Dentistry, IRCCS Associazione Oasi Maria SS, Troina (EN), Italy; 2 Unit of Pedagogy, IRCCS Associazione Oasi Maria SS, Troina (EN), Italy; 3 Laboratory of Molecular Genetics, Unit of Medical Genetics, IRCCS AssociazioneOasi Maria SS, Troina (EN), Italy

**Keywords:** Dental anxiety scale, Dental fear, Intellectual disabilities

## Abstract

**Background:**

This study was aimed to investigate the prevalence of dental anxiety in a population of patients with Borderline Intellectual Functioning (BIF) and patients with mild and moderate intellectual disability (ID), and how dental anxiety correlated with their age and gender.

**Methods:**

The sample was made of 700 patients, 287 females and 413 males, 6-to-47 years old, either with borderline intellectual functioning or mild/moderate intellectual disabilities. All patients were administered the Dental Anxiety Scale to assess their level of dental anxiety.

**Results:**

Moderate Anxiety was the most prevalent dental anxiety category for patients with intellectual borderline functioning (15.56 %) and mild intellectual disabilities(18.79 %), while Severe Anxiety was the most prevalent category for patients with moderate intellectual disabilities(21 %). Overall, a statistically significant difference (*p* < 0.001) between the three groups (BIF, Mild-ID and Moderate-ID) was found. Also, the correlation analysis between participants’ age and dental anxiety was statistically significant (*p* < 0.001); indeed, dental anxiety turned out to decrease with the increasing of the age. Moreover, the analysis between gender and dental anxiety was found to be significant as well (*p* < 0.001), where higher prevalence of dental anxiety was found in females.

**Conclusions:**

To our knowledge, this is the first study on dental anxiety carried out in the field of intellectual disability. Results show that the higher the level of intellectual disability – and consequently the lower the cognitive functioning – the higher the percentage and the severity of dental anxiety.

## Background

Despite the continuous evolution of dental technologies, dental anxiety has been consistently reported for long time [1]. Dental anxiety, fear and phobia are subcategories of overall anxiety [2].

The level of anxiety and fear when undergoing dental treatments in the general population has been investigated in many countries. In France, Nicolas et al. [3] studied the percentage and severity of dental anxiety in the adult population, using a French version of the Dental Anxiety Scale (DAS). Other countries have also performed studies on dental anxiety, such as Denmark [4], England [5], Finland [6], Saudi Arabia [7], Hungary [8], and Sri Lanka [9]. Several studies have attempted to determine a correlation between fear of the dentist and other factors, in order to prevent some anxiety causes, to recognize anxious subjects and design customized treatments. The variables most often considered to be associated to anxiety were: gender, age, level of education, past dental experiences, fear of pain, parents’ attitudes and influence. Results from the previous literature have not always been consistent, albeit the majority of authors agree that anxiety toward dental care is one of the major causes of renouncing dental treatment, thus impacting on social and economic aspects.

A higher prevalence of anxiety in females was found in Turkey, in the studies by Firat et al. [10] and Tunc et al. [11], and in India in the study by Mohammed et al. [1]. In Australia as well, Armfield et al. [12] found that the fear of the dentist was prevalent in females and correlated with the age of the sample. Nevertheless, Erten at al. [13] found no statistically significant differences between genders and no meaningful effects of the age on the final results, while a significant correlation with the level of education and the frequency of dental care sessions was reported.

In India, Appukuttan et al. [14] reported a correlation between dental anxiety and painful dental care experiences. In Brazil, the study by Soares et al. [15] aimed to determine anxiety prevalence in dentistry practice by analyzing correlations between previous experiences and history of pain. Honkala et al. [16] measured dental anxiety prevalence in adolescents from Kuwait using the Modified Dental Anxiety Scale (MDAS), whereas Majstorovic et al. [17] compared children’s and parents’ fears.

Finally, other authors, such as Liddel and Locker [18], in 1997, reported a higher level of dental anxiety in females and young persons than in males and older persons. The same authors, however, stated that the main factor playing a role in determining dental anxiety was the fear of pain, which seems to be more prevalent in males than in females.

Different studies (e.g. Reid et al.) [19], have observed that intellectual disabilities relate with anxiety disorders, therefore, our aim was to investigate how specific anxiety (dental anxiety) relates to ID. In the field of intellectual disabilities (ID), because of the high prevalence of marked functional impairments and psychiatric disorders, the fear of the dentist makes the dental treatment more difficult.

Not many studies have dealt with dental anxiety rehabilitation interventions in the ID population and, to our knowledge, there are no available estimates of its prevalence in this population.

The aim of this study is to investigate dental anxiety and its correlation with age and gender of patients with Borderline Intellectual Functioning (BIF) as well as patients with mild and moderate Intellectual Disabilities (Mild-ID; Moderate-ID respectively). See Table 1 for a detailed description of the different kinds of ID.

**Table 1:** Borderline Intellectual Functioning and mild/moderate intellectual disabilities

*Borderline Intellectual Functioning (BIF)*
Borderline Intellectual Functioning is the result of different causes. In the DSM-5, IQ boundaries are no longer part of the classification: a careful assessment of intellectual and adaptive functions and their discrepancies is required, although a clear definition of the disorder is not provided. In the previous versions of the DSM, intellectual functions were measured with IQ testing procedures and cognitive functioning was defined as an IQ range that is higher than that for mental retardation – cut-off value: 1–2 standard deviations from the mean (IQ 71–84).
*Mild Intellectual Disability (Mild-ID)*
Individuals with Mild intellectual disability present with difficulties in conceptual domains such as, learning academic skills, time and money, with support needed in one or more areas to meet age-related expectations. In adults, abstract thinking, executive function and short-term memory are impaired. In the social domain, individuals are immature in social interactions. Communication, conversation and language are more concrete than expected for age. In the practical domain, individuals need some support with complex daily living tasks. In the previous versions of DSM, the IQ fell between 50–55 and 70.
*Moderate Intellectual Disability (Moderate-ID)*
In Moderate intellectual disability, progress in conceptual skills occurs slowly especially in language development, academic skills, understanding of time and money, which result markedly limited compared with that of peers and thus requiring continuous support. In the social domain, despite a certain capacity for relationships, social judgment and decision-making abilities are limited. In the practical domain, individuals can care for personal needs; independent employment can be achieved only in jobs that require limited conceptual and communication skills. Maladaptive behaviour is present in a significant minority and causes social problems. In the previous versions of the DSM, the IQ ranged from 35–40 through 50–55.

## Methods

### Sample

The study was approved by the Ethical Committee of the Research Institute “IRCCS Associazione Oasi Maria SS.”, Troina (EN), Italy. Written informed consent was obtained for all participants. The subjects were recruited among 734 consecutive patients referring to diagnostic and rehabilitation services of the IRCCS Associazione Oasi Maria SS. (Troina – EN – Italy), a research institute dealing with treatment and rehabilitation in the field of intellectual disabilities. All participants with BIF and ID were diagnosed by expert qualified psychologists, following the DSM-5 (2014) criteria [20] (See also Table 1). Thirty-four subjects were unable to complete the questionnaire because of their low intellectual level; thus, the final sample consisted of 700 Italian patients, 287 females and 413 males. Their age ranged from 6 to 47 years. The sample was divided into three subgroups: 270 patients with BIF, 330 with Mild-ID, and 100 with Moderate-ID.

### Questionnaire

Dental anxiety was assessed using Corah’s DAS, 1978 [21]. It’s a four-item scale with 5 multiple-choice answers, tapping on dental practice-related situations and assessing anticipatory anxiety as well as assumed anxiety during treatment (Table 2). Answers were scored from 4 to 20 and grouped in 5 categories: No Anxiety (NA, score = 4); Slight Anxiety (SLA, score = 5 to 8); Moderate Anxiety (MA, score = 9 to 12); High Anxiety (HA, score = 13 to 14); Severe Anxiety (SA, score = 15 to 20). Personal data were collected and integrated to the information derived from the scale. Corah’s scale was administered through interviews either to patients. Data were collected between January 2014 and May 2015. Our a priori hypotheses were that, in our ID sample, anxiety levels would show differences depending on the gender and age variables.

**Table 2:** Dental Anxiety Scale questionnaire (by Corah et al. 1978) [21]

1. If you had to go to the dentist tomorrow, how would you feel about it?
✓I would look forward to it as a reasonably enjoyable experience.
✓I wouldn’t care one way or the other.
✓I would be a little uneasy about it.
✓I would be afraid that it would be unpleasant and painful.
✓So anxious, that I sometimes break out in a sweat or almost feel physically sick.
2. When you are waiting in the dentist’s office for your turn in the chair, how do you feel?
✓Relaxed.
✓A little uneasy.
✓Tense.
✓Anxious
✓So anxious, that I sometimes break out in a sweat or almost feel physically sick.
3. When you are in the dentist’s chair waiting while he gets his drill ready to begin work on your teeth, how do you feel?
✓Relaxed.
✓A little uneasy.
✓Tense.
✓Anxious
✓So anxious, that I sometimes break out in a sweat or almost feel physically sick.
4. You are in the dentist’s chair to have your teeth cleaned. While you are waiting and the dentist is getting out the instruments which he will use to scrape your teeth around the gums, how do you feel?
✓Relaxed.
✓A little uneasy.
✓Tense.
✓Anxious.
✓So anxious, that I sometimes break out in a sweat or almost feel physically sick

### Statistical analyses

**Fig. 1:**
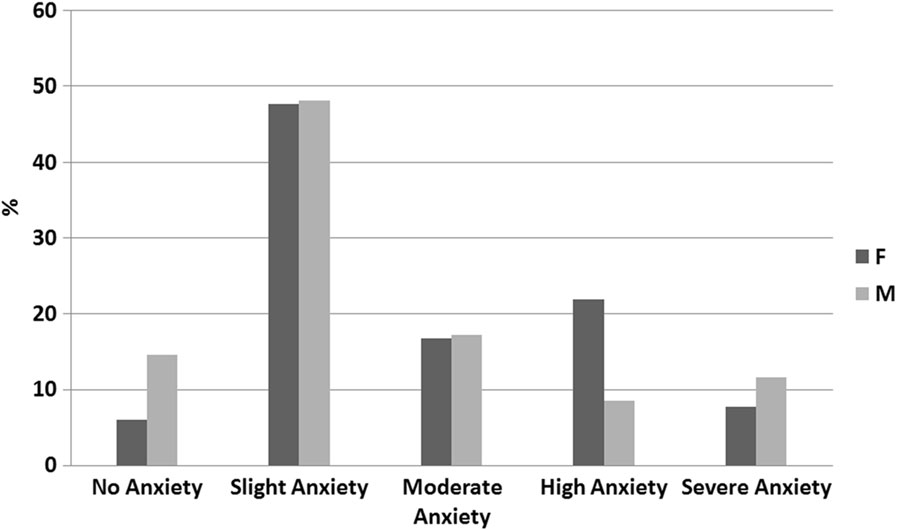
Anxiety rating (DAS scale) in males/females comparison

For the statistical analyses, the *chi-square* and Pearson’s correlation tests were used. *p* values less than 0.05 were considered to be statistically significant. The statistical appropriateness of the chi-square tests used in this study was assessed “*a posteriori*” by calculating first the effect size of each test carried-out and then calculating the corresponding sample size required with α = 0.05, resulting in an actual power of 98 % and a required sample size of 399 for the 5 × 3 matrix (Table 3) and of 202 for the 5 × 2 matrix (Fig. 1).

**Table 3:** Anxiety rating in patients with Borderline Intellectual Functioning (BIF) and patients with Intellectual Disabilities (IDs)

	BIF		Mild-ID		Moderate-ID	
Anxiety Rating	N	%	N	%	N	%
No Anxiety (NA)	26	9.63	40	12.12	11	11
Slight Anxiety (SLA)	168	62.22	134	40.61	34	34
Moderate Anxiety (MA)	42	15.56	62	18.79	15	15
High Anxiety (HA)	21	7.78	58	17.58	19	19
Severe Anxiety (SA)	13	4.81	36	10.91	21	21
Total	270	100	330	100	100	100

*N* Number of patients

## Results

Seven hundred Italian participants were analyzed for this study, 287 females and 413 males, with an age range of 6-to-45 years for females (mean age: 18.51, +/− 9.84 SD) and 6-to-47 for males (mean age: 16.93, +/− 8.99 SD). Results obtained from the three subgroups (BIF, Mild-ID and Moderate-ID) are shown in the Table 3.

The highest percentages of the three sample subgroups fell within the SLA range. These percentages decreased with the increase in severity level of IDs. As far as the BIF group is concerned, 15.56 % of patients fell within the MA range, 7.78 % within the HA range and 4.81 % within the SA range. As for the Mild-ID group, the highest percentage was found in the MA range (18.79 %, higher than in BIF); 17.58 % (doubling the BIF) fell within the range of HA range. Finally, the percentage peak for the group with Moderate-ID fell within the SA range (21 %), followed by the HA range (19 %).

**Fig. 2:**
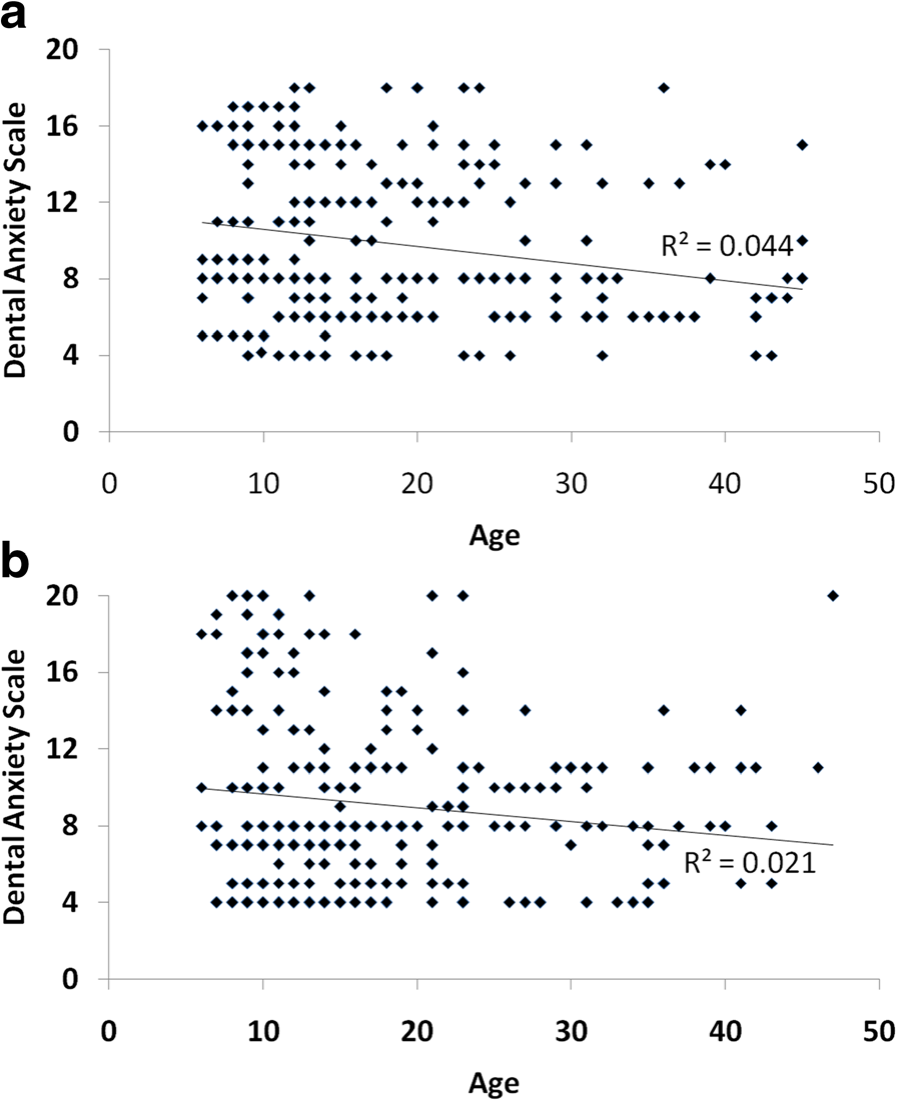
Scatterplot of the relationship between Dental Anxiety Scale and Age in females (**a**) and males (**b**). Trend line and R^2^ values are shown

Overall, a statistically significant difference (*chi-square* = 53.14, *p* < 0.001) between the three groups (BIF, Mild-ID and Moderate-ID) was found using chi-square test. The correlation analysis (Pearson’s *coefficient*) between participants’ age and dental anxiety was statistically significant (*r* = −0.166, *p* < 0.001). When the data were analyzed separately by gender it was statistically significant (males: *r* = −0.145, *p* < 0.002; females: *r* = −0.209, *p* < 0.001; see Fig. 2). Fig. 1 shows results from DAS, comparing data obtained by gender, which were found to be significant (*chi-square* = 36.044, *p* < 0.001) using chi-square test. Within the NA range, male patients turned out to be the most numerous. Within the HA, the chart shows how the female percentage was higher than that of males.

## Discussion

The higher the ID, as shown in Table 3, the lower the cognitive functioning and the higher the percentage of severity of dental anxiety.

To our knowledge, this is the first epidemiological study on dental anxiety in a population with intellectual disabilities, hence the incomparability of our data with those obtained from other authors. However, we have estimated that the increase in dental anxiety, directly proportional to higher levels of intellectual disability, might correlate with the fact that this population has lower psychological resources available to effectively face stressing events, is deprived of several cognitive abilities such as memory, problem-solving and planning skills [22]. As far as phobias are concerned, they seem to be prevalent in individuals with ID. Moreover, a number of phobias are physiologically related to age, therefore some kind of fears in ID individuals might be relating to their developmental levels [23, 24].

The significant correlation that was found in this study between the age of the sample and dental anxiety highlights that decreased levels of dental anxiety can be detected more in the older than the younger population. Note that significant correlation didn’t change when analysis was performed separately by gender (Fig. 2). In Australia, Armfield et al. [12] carried out a telephone survey with a sample of 7,312 individuals 5 to over 80 years old. The highest prevalence of fear was found in the adult population 40–64 years old, while the lowest in the over-80. These results seem also to confirm a correlation between age and dental anxiety, but are inconsistent with our findings. However, it’s worth noting that our sample’s age ranges largely differed from Armfield’s.Erten et al. [13] administered the DAS questionnaire and the Dental Fear Scale (DFS) to 1,437 patients, divided into 5 groups by age. Authors reported no statistically significant difference between genders and the correlation between dental anxiety and the age of patients did not significantly affected final results; on the other hand, a significant correlation was found with the level of education and frequency of dental treatments of patients.

Further investigations focusing on the frequency of dental treatments and their correlation with age are strongly recommended in future studies, in order to confirm our hypothesis that the older the sample, the higher the frequency of dental treatments and the lower the dental anxiety.

In their Indian study, Mohammed et al. [1] examined a sample of 340 individuals, 180 females and 160 males, 15–65 years old. Results from DAS questionnaire showed high levels of anxiety in the age range 25–35, and low levels of anxiety in the age range 55–65. This finding is consistent with our results. Appukuttan et al. [14] examined a sample of 1,148 patients 18–70 years old, who were administered the MDAS questionnaire to assess their level of anxiety. Younger patients turned out to be more anxious, while dental anxiety seemed to decrease with the age.

Locker and Liddel in 1991 [25] reported the capability of facing certain events based on previous experiences, and the capability of adapting to unavoidable conditions, as relevant factors in reducing dental anxiety; in 1997, these authors [18] also stated that gender and age ranges seemed to be reflected into attitudes towards pain.

Another important finding in our study was that anxiety seemed to be more prevalent in females than in males. Male patients turned out to be the most numerous within the NA range, while within the HA the female percentage was higher than that of males. (see Fig. 1). These statistically significant data are consistently with findings by Armfield et al. [12], Firat et al. [10], Tunc et al. [11], Liddel et al. [18], Mohammed et al. [1], Majstorovic et al. [17], and Appukuttan et al. [14].

The samples recruited in the studies above are largely variable as for the number of participants and the age ranges; nevertheless, the majority of them seem to indicate a prevalence of dental anxiety in female samples.

Honkala et al. [16] in Kuwait found that, in a sample of 757 students 13–15 years old, one third of the girls and 6 % of the boys were afraid of visiting a dentist.

Soares et al. [15] in Brazil, in a sample of 101 children 6–16 years old, found that female children turned out to be three times more anxious than male children.

It might be that the higher prevalence of dental anxiety in female samples is due to women’s willingness to overtly admit their fears. Our hypothesis of a prevalence of dental anxiety in female samples is also corroborated by other authors [26–29]. A potential variable for further investigate would be personality styles. Since a correlation exists between personality styles and anxiety levels [30], in future studies, it would be interesting to analyze how specific anxiety (dental anxiety) relates to personality styles.

## Conclusions

To our knowledge, this is the first study on dental anxiety carried out in the field of ID. Based on our results, the higher the ID – and therefore the lower the cognitive functioning – and the higher the percentage and severity of dental anxiety. Moreover, the age of the sample significantly correlated with patients’ dental anxiety levels. Finally, higher percentages of dental anxiety were found in the female portion of the sample.

Based on our findings, further investigations might include other variables, such as past traumatic dental experiences, and the number of exposures to dental treatments.

## Abbreviations

BIF: Borderline intellectual functioning
DAS: Dental anxiety scale
DFS: Dental fear scale
HA: High anxiety
ID: Intellectual disabilities
MA: Moderate anxiety
MDAS: Modified dental anxiety scale
Mild-ID: Mild intellectual disabilities
Moderate-ID: Moderate intellectual disabilities
NA: No anxiety
SA: Severe anxiety
SLA: Slight anxiety
